# Genome-wide identification of nuclear receptor (NR) superfamily genes in the copepod *Tigriopus japonicus*

**DOI:** 10.1186/1471-2164-15-993

**Published:** 2014-11-18

**Authors:** Dae-Sik Hwang, Bo-Young Lee, Hui-Su Kim, Min Chul Lee, Do-Hyun Kyung, Ae-Son Om, Jae-Sung Rhee, Jae-Seong Lee

**Affiliations:** Department of Biological Science, College of Science, Sungkyunkwan University, Suwon, 440-746 South Korea; Department of Food and Nutrition, College of Human Ecology, Hanyang University, Seoul, 133-791 South Korea; Department of Chemistry, College of Natural Sciences, Hanyang University, Seoul, 133-791 South Korea; Department of Marine Science, College of Natural Science, Incheon National University, Incheon, 406-772 South Korea

**Keywords:** Nuclear receptor, Copepod, Ecdysone signaling

## Abstract

**Background:**

Nuclear receptors (NRs) are a large superfamily of proteins defined by a DNA-binding domain (DBD) and a ligand-binding domain (LBD). They function as transcriptional regulators to control expression of genes involved in development, homeostasis, and metabolism. The number of NRs differs from species to species, because of gene duplications and/or lineage-specific gene losses during metazoan evolution. Many NRs in arthropods interact with the ecdysteroid hormone and are involved in ecdysone-mediated signaling in arthropods. The nuclear receptor superfamily complement has been reported in several arthropods, including crustaceans, but not in copepods. We identified the entire NR repertoire of the copepod *Tigriopus japonicus*, which is an important marine model species for ecotoxicology and environmental genomics.

**Results:**

Using whole genome and transcriptome sequences, we identified a total of 31 nuclear receptors in the genome of *T. japonicus*. Nomenclature of the nuclear receptors was determined based on the sequence similarities of the DNA-binding domain (DBD) and ligand-binding domain (LBD). The 7 subfamilies of NRs separate into five major clades (subfamilies NR1, NR2, NR3, NR4, and NR5/6). Although the repertoire of NR members in, *T. japonicus* was similar to that reported for other arthropods, there was an expansion of the NR1 subfamily in *Tigriopus japonicus*. The twelve unique nuclear receptors identified in *T. japonicus* are members of NR1L. This expansion may be a unique lineage-specific feature of crustaceans. Interestingly, E78 and HR83, which are present in other arthropods, were absent from the genomes of *T. japonicus* and two congeneric copepod species (*T. japonicus* and *Tigriopus californicus*), suggesting copepod lineage-specific gene loss.

**Conclusions:**

We identified all NR receptors present in the copepod, *T. japonicus*. Knowledge of the copepod nuclear receptor repertoire will contribute to a better understanding of copepod- and crustacean-specific NR evolution.

**Electronic supplementary material:**

The online version of this article (doi:10.1186/1471-2164-15-993) contains supplementary material, which is available to authorized users.

## Background

Nuclear receptors (NRs) are a group of proteins defined by the presence of two functional domains: a highly conserved DNA-binding domain (DBD) and a less conserved ligand-binding domain (LBD). They function as transcriptional regulators to control the expression of specific genes involved in development, homeostasis, and metabolism [1]. Nuclear receptors are a large superfamily specific to metazoans, and seven subfamilies (NR0 to NR6) are currently recognized based on structural and functional data [2]. The superfamily includes receptors for hydrophobic molecules such as steroid hormones, retinoic acids, thyroid hormones, and fatty acids [3]. Two large subfamilies are NR1 and NR2. The subfamily NR1 includes the thyroid hormone receptors (TRs), retinoic acid receptors (RARs), vitamin D receptors (VDRs), and peroxisome proliferator-activated receptors (PPARs). NR2 subfamily contains retinoid X receptors (RXRs) and hepatocyte nuclear factor 4 (HNF4). NR3 subfamily comprises receptors for sex and adrenal steroid hormones. While steroid hormone receptors (SRs) such as the androgen receptor (AR), estrogen receptor (ER), glucocorticoid (GR), mineralocorticoid (MR), and progestagen receptor (PR) belong to NR3, the insect SR, ecdysone receptor (EcR) is in the NR1 clade [4]. Some NRs are referred to as orphan nuclear receptors because their endogenous ligands remain unknown [5]. Subfamily NR4 contains orphan receptors such as nerve growth factor-induced clone B (NGFIB), nuclear receptor related 1 (NURR1), and insect HR38 [6–8]. Subfamily NR5 includes Fushi Tarazu-factor 1 (FTZ-F1) and HR39, which are involved in ecdysone-regulated response pathways [9–11]. NR6 contains insect HR4, which is homologous to the vertebrate orphan receptor germ cell nuclear factor (GCNF). The last subfamily, NR0, includes two groups, A and B. NR0A group members lack a LBD and have been found only in insects, while members of NR0B, DAX1 and SHP, have been identified only in vertebrates [1]. Because nuclear receptors are dispersed in genomes and functionally well characterized, they are considered a good model to study gene or genome duplication [12]. Genome sequencing and comparative genomic studies have enabled identification of all nuclear receptor family members in some organisms. The number of NRs identified in sequenced genomes varies from species to species, as some nuclear receptors have undergone several gene duplication events during metazoan evolution [12] while others have been lost due to gene loss. There are an estimated 21 NRs in *Drosophila melanogaster*, 25 in *Daphnia pulex*, 21 in *Tribolium castaneum*, 48 in *Homo sapiens*, and 284 in *Caenohabditis elegans* [13–17]. An interesting feature of nuclear receptors in arthropods is that several nuclear receptors interact with the ecdysteroid hormone and are involved in ecdysone-mediated signaling, which regulates development, growth, and molting during embryogenesis [10, 18]. Molting and metamorphosis in arthropods are remarkable developmental processes trigger by ecdysteroid hormones and signal transduction by members of the nuclear receptor superfamily. During the onset of metamorphosis, ecdysone regulates expression of several members of the NR superfamily [19, 20]. NRs, therefore, are considered to be responsible for the evolution of insect metamorphosis by responding to ecdysone and juvenile hormone [20]. Insect maturation is regulated by the steroid hormone 20-hydroxyecdysone (20E), which binds to a heterodimer of EcR and ultraspiracle (USP) [21]. In addition, many nuclear receptors such as DHR3, DHR4, DHR39, E75, E78, and FTZ-F1 function as direct targets of 20E-EcR-USP [10, 22–24]. DHR38 functions as a second ecdysteroid receptor by dimerizing with USP or RXR, while DHR78 inhibits 20E-induced reporter gene transcription by binding to a subset of ECR-USP binding sites in cultured cells [25, 26]. ERR directs a metabolic switch that supports developmental growth [27].

The copepod *Tigriopus* is a marine model species for ecotoxicology and environmental genomic studies [18]. Copepoda is the second largest crustacean taxa and one of the dominant taxa in aquatic zooplankton communities, representing 70% of the ocean’s zooplankton biomass [28, 29]. The nuclear receptor superfamily complement of some arthropods, including crustaceans, has been reported, but not that of any copepods. In this study, we identified the complete nuclear receptor repertoire of the copepod *T. japonicus* based on whole genome sequence and transcriptome data [30].

## Results

### Identification of nuclear receptors in *T. japonicus*

**Figure 1:**
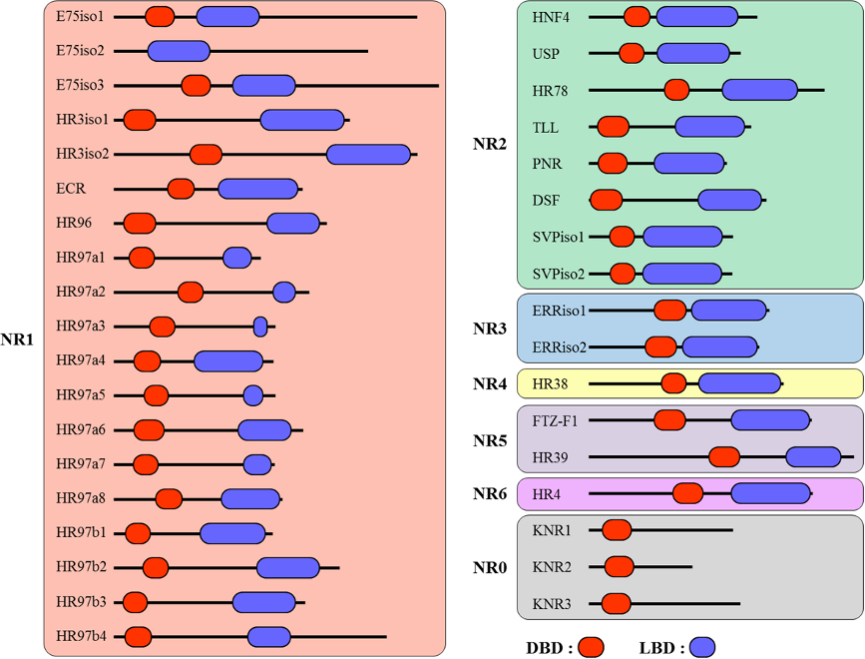
**Schematic representation of the DBD and LBD domains in each nuclear receptor identified in** ***T. japonicus*** **.** Amino acid sequences of entire NRs are drawn to scale. DNA-binding domains (DBDs) and ligand-binding domains (LBDs) are colored in red and blue, respectively. Isotypes of each NR are abbreviated with ‘iso’.

Using whole genome sequences and RNA-seq sequences of *T. japonicus*, we performed a BLAST analysis against the non-redundant (NR) database of NCBI to obtain contigs containing the DBD and/or LBD domains of nuclear receptors. We found 67 contigs containing full or partial nuclear receptor DBD sequences. Of these 67 contigs, 42 had both DBD and LBD sequences of nuclear receptors. Full-length sequences of the putative nuclear receptors were obtained from partial sequences using RACE (Rapid Amplification of cDNA Ends) methods. Finally, full-length mRNAs from 36 contigs were confirmed by RT-PCR analysis (Additional file 1: Figure S1; Additional file 1: Table S1). Further analysis of a conserved domain database allowed us to determine the DBD and LBD regions of each nuclear receptor [31] and revealed that four receptors (NR1D TJ-E75, NR1F TJ-HR3, NR2F TJ-SVP, and NR3B TJ-ERR) had more than two isoform structures (Figure 1, Additional file 1: Figure S2). TJ-E75A and TJ-E75C shared both the DBD and LBD, while TJ-E75B shared only the LBD (Figure 1, Additional file 1: Figure S2A). In the case of TJ-HR3, two isoforms shared both the DBD and LBD (Figure 1, Additional file 1: Figure S2B). The two isoforms of TJ-SVP have an independent DBD and LBD in a consecutive tail to head structure (Figure 1, Additional file 1: Figure S2C). Two TJ-ERR isoforms shared both a DBD and LBD (Figure 1, Additional file 1: Figure S2D). Nucleotide sequences of each isoform were confirmed by RT-PCR validation (Additional file 1: Figure S1). We thus found a total of 31 nuclear receptors in the *T. japonicus* genome; the sequences of these receptors have been deposited in GenBank (Table 1).

**Table 1:** **Details of nuclear receptors identified in** ***T. japonicus***

Subfamily	Group	Nuclear receptors	Size (amino acids)	Accession no. (GenBank ID)	Homologous receptors (Arthropods/Chordates)
NR1	D	TJ-E75a-iso1	897	KJ664189	E75/Rev-Erb-a, Rev-Erb-b
		TJ-E75b-iso2	752	KJ664190	
		TJ-E75c-iso3	1004	KJ664191	
	F	TJ-HR3-iso1	688	KJ664192	HR3/RORa, RORb, RORg
		TJ-HR3-iso2	883	KJ664193	
	H	TJ-ECR	546	ADD82902	EcR/LXRa, LXRb, FXR
	J	TJ-HR96	630	KJ664194	HR96/-
	L	TJ-HR97a1	418	KJ664195	HR97/-
		TJ-HR97a2	566	KJ664196	
		TJ-HR97a3	482	KJ664198	
		TJ-HR97a4	448	KM676402	
		TJ-HR97a5	467	KJ664199	
		TJ-HR97a6	560	KJ664201	
		TJ-HR97a7	476	KJ664206	
		TJ-HR97a8	499	KJ664205	
		TJ-HR97b1	470	KJ664200	
		TJ-HR97b2	668	KJ664202	
		TJ-HR97b3	566	KJ664203	
		TJ-HR97b4	807	KJ664205	
NR2	A	TJ-HNF4	498	KJ664207	HNF4/HNF4a, HNF4g
	B	TJ-USP	449	KJ664208	USP/RXRa, RXRb, RXRg
	D	TJ-HR78	697	KJ664209	HR78/-
	E	TJ-TLL	480	KJ664210	TLL/TLX
		TJ-PNR	408	KJ664211	HR51, PNR/PNR
		TJ-DSF	525	KJ664212	DSF/-
	F	TJ-SVP-iso1	424	KJ664213	SVP/COUP-TFa, COUP-TFb
		TJ-SVP-iso2	426	KJ664214	
NR3	B	TJ-ERR-iso1	504	KJ664215	ERR/ERRa, ERRb, ERRg
		TJ-ERR-iso2	534	KJ664216	
NR4	A	TJ-HR38	589	KJ664217	HR38/NGF1B, NURR1, NOR1
NR5	A	TJ-FTZ-F1	659	KJ664218	FTZ-F1/SF1, LRH1
	B	TJ-HR39	784	KJ664219	HR39/-
NR6	A	TJ-HR4	662	KJ664220	HR4/GCNF
NR0	A	TJ-KNR1	426	KJ664221	KNI, KNRL, EGON/-
		TJ-KNR2	306	KJ664222	
		TJ-KNR3	448	KJ664223	

Both the arthropod names and the official nomenclature names for chordates are given for each nuclear receptor. Name of isotypes for each NR are abbreviated with ‘iso’.

### Phylogenetic analysis and nomenclature

**Figure 2:**
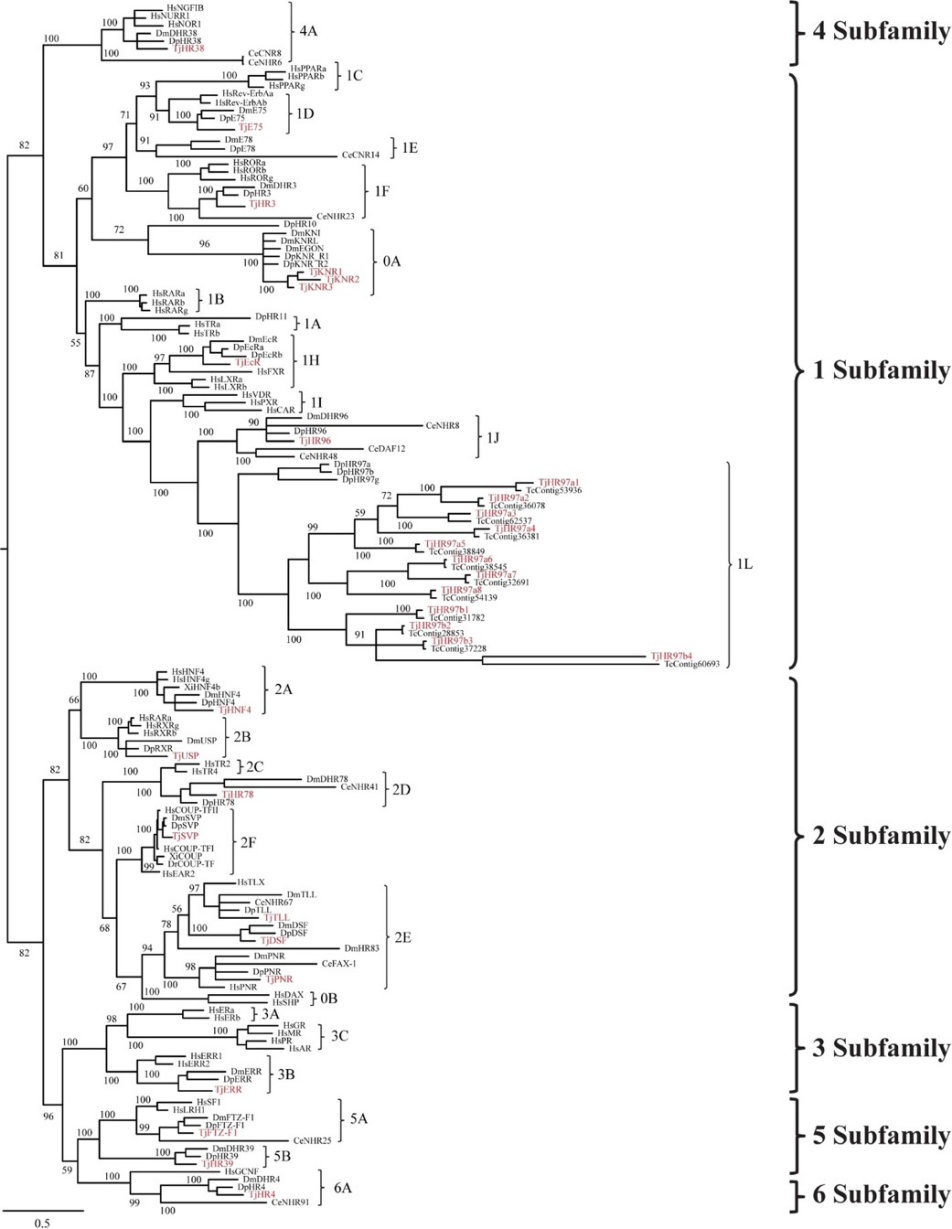
**Gene phylogeny of nuclear receptors in** ***T. japonicus*** **and other related species.** Phylogenetic distance was calculated with combined DBD-LBD amino acid sequences from *T. japonicus* and other species. A best-fit substitution model was established using maximum likelihood (ML) analysis supported by MEGA (ver.6.0). Numbers at nodes represent ML bootstrap support values and Bayesian posterior probabilities (=1.00). Details of model testing and parameters are provided in the Methods section. Tree is proportionally scaled, with the scale bar indicating sequence distance as number of substitutions. Species abbreviations: Ce: *Caenorhabditis elegans*, Dm: *Drosophila melanogaster*, Dp: *Daphnia pulex*, Dr: *Danio rerio*, Gg: *Gallus gallus*, Hs: *Homo sapiens*, Tj: *Tigriopus japonicus*, Xl: *Xenopus laevis*.

**Figure 3:**
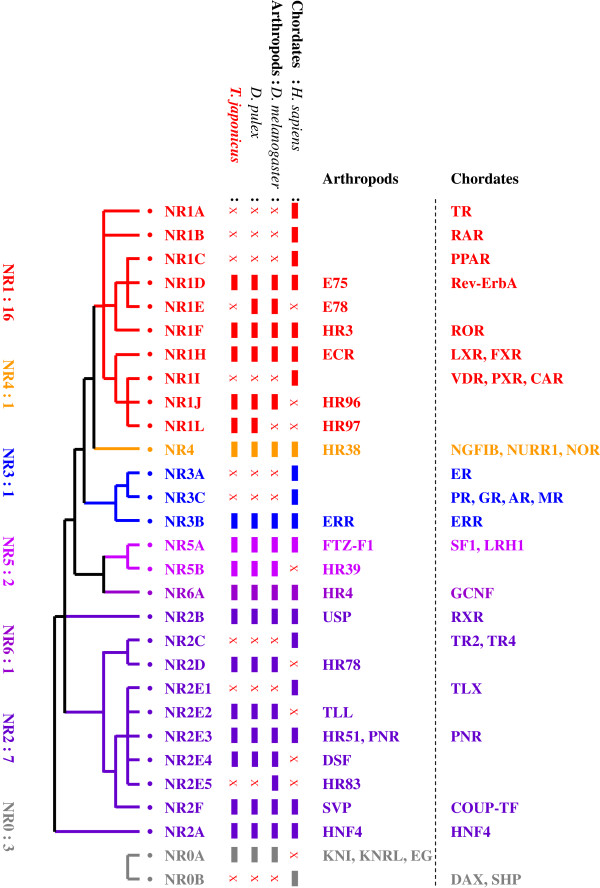
**Simplified phylogenetic distribution of nuclear receptors in chordates and arthropods.** This diagram was updated based on schematic features of the NR superfamily suggested by a previous study [34]. Both arthropod names and the official nomenclature names of chordates are given for each nuclear receptor. A colored box indicates the presence of a homolog, while the character X indicates the absence of the NR from the genome of that species.

Phylogenetic analysis of nuclear receptors of *T. japonicus* and sequence comparisons with other organisms (*C. elegans*, *D. pulex*, *D. melanogaster*, and *H. sapiens*) were performed to classify the nuclear receptors found in *T. japonicus* and to investigate their evolutionary history with maximum likelihood method. The phylogenetic tree segregated the nuclear receptors of *T. japonicus* into five major clades (subfamily NR1, NR2, NR3, NR4, and NR5/6) (Figure 2). Seven subfamilies with 16 groups were evident (Table 1). Of the 31 nuclear receptors we identified, 16 receptors belonged to five groups (D, F, H, J, and L) of subfamily NR1, seven NRs to five groups (A, B, D, E, and F) of subfamily NR2, two NRs to two groups (A and B) of subfamily NR5, and three NRs to group A of NR0 (Table 1). Subfamilies NR3, NR4, and NR6 contained one member in each subfamily. Overall repertoire of NR subfamily groups in *T. japonicus* followed that seen in other arthropods, which is distinct from that in *H. sapiens* as an example of a chordate, although NR sets also vary between different vertebrate species [32] (Figure 3). Non-SRs such as TR, RAR, and PPAR in NR1 and SRs such as ER and members in NR3C have not been detected in arthropods, as represented by *D. melanogaster* and *D. pulex* [17, 33]. Similarly, the NR2E1 group in the NR2E subfamily is present in *H. sapiens* but not arthropods or crustaceans, whereas the opposite pattern has been observed for members of the NR2E2 and NR2E4 groups.

### Nuclear receptor features unique to *T. japonicus*

Of the 16 nuclear receptors in subfamily NR1, 12 members (TJ-HR97a1, TJ-HR97a2, TJ-HR97a3, TJ-HR97a4, TJ-HR97a5, TJ-HR97a6, TJ-HR97a7, TJ-HR97a8, TJ-HR97b1, TJ-HR97b2, TJ-HR97b3, and TJ-HR97b4) were unique to *T. japonicus* (Table 1). Phylogenetic analysis placed the 12 NRs together as the sister group to a clade of NR1L receptors identified in *D. pulex* (abbreviated in previous studies as Dappu- or D) (DHR97a, DHR97b, and DHR97g) (Figure 2) [13–17]. We assigned 12 NRs to HR97a1-8, and HR97b1-4, because the NR1L (HR97) group was recently characterized in *Daphnia* species [17, 35], although the 12 members were formerly duplicated from *D. melanogaster* and *D. pulex* HR96 (NR1J) with an independent lineage-specific expansion of three HR97 isoforms in *D. pulex* (Table 1). The genetic distance of the *T. japonicus* NR1L members to the other NR1 groups was relatively high (Tables 2, 3 and 4). Moreover, distance values of NR1La (0.797) and NR1Lb (0.871) in *T. japonicus* from other NR1L groups were higher than all *Daphnia* NR1L members (i.e. NR1La, NR1Lb, and NR1Lg; 0.324) (Table 4). Sequence similarity of the DBD and LBD of the 12 receptors and DHR97 ranged from 50 to 70% for the DBD and 9 to 37% for the LBD (Additional file 1: Table S2). The range of DBD sequence similarities among the 12 members in group NR1L was 43 to 78% (Additional file 1: Table S3).

**Table 2:** **Estimation of evolutionary distances of sequence pairs comprising** ***T. japonicus*** **NR1L subfamily members with other NR1 subfamilies: A) Genetic distance values among NR1I, NR1J, and all NR1L members**

	in group	NR1I	NR1J	NR1L
NR1I	0.379		0.807	1.365
NR1J	0.479			1.328
NR1L^1^	1.063			

^1^Entire members of NR1L subfamilies.

Genetic distance is represented as the number of amino acid substitutions per site averaged over all sequence pairs between groups. Details of the calculations and parameters are provided in the Methods section. Raw results are appended as Additional file 1: Table S4. Gene information for NR1I, NR1J, and NR1L was collected from *Caenorhabditis elegans* (Ce), *Ciona intestinalis* (Ci), *Danio rerio* (Dr), *Daphnia magna* (Dma), *Daphnia pulex* (Dp), *Drosophila melanogaster* (Dm), *Homo sapiens* (Hs), *Ixodes scapularis* (Is), *Schistosoma mansoni* (Sm), *Tigriopus californicus* (Tc), and *Xenopus laevis* (Xl).

**Table 3:** **Estimation of evolutionary distances of sequence pairs comprising** ***T. japonicus*** **NR1L subfamily members with other NR1 subfamilies: B) Genetic distance values among NR1I, NR1J,** ***Daphnia*** **NR1L, and copepod NR1L members**

	in group	NR1I	NR1J	NR1L	NR1La
NR1I	0.379		0.807	0.949	1.492
NR1J	0.479			1.002	1.427
NR1L^1^	0.324				1.239
NR1L^2^	1.013				

^1^Entire *Daphnia* NR1L subfamily.

^2^All copepod NR1L subfamilies.

**Table 4:** **Estimation of evolutionary distances of sequence pairs comprising** ***T. japonicus*** **NR1L subfamily members with other NR1 subfamilies: C) Genetic distance values among NR1I, NR1J,** ***Daphnia*** **NR1L, and copepod NR1La and NR1Lb members**

	in group	NR1I	NR1J	NR1L	NR1La	NR1Lb
NR1I	0.379		0.807	0.949	1.579	1.435
NR1J	0.479			1.002	1.537	1.357
NR1L^1^	0.324				1.250	1.231
NR1La^2^	0.797					1.178
NR1Lb^3^	0.871					

^1^Entire *Daphnia* NR1L subfamily.

^2^All copepod NR1La subfamilies.

^3^All copepod NR1Lb subfamilies.

This kind of gene expansion in NR1L has also been observed in other copepods; 12 NR1L members have been identified in *Tigriopus californicus* (Prof. Ronald S. Burton, *T. californicus* Transcriptome Shotgun Assembly; http://www.ncbi.nlm.nih.gov/nuccore/?term=txid6832%5BOrganism%3Anoexp%5D+AND+TSA) and eight NR1L members have been identified in the copepod *Paracyclopina nana* (Prof. Jae-Seong Lee, unpublished data). These NR1L members are considered unique to crustaceans, as the NR1L group has been identified in *Daphnia* species and three copepods, but not in arthropods. As described above, *T. japonicus* had a similar set of nuclear receptors to those present in other arthropods (Figure 3). However, we were not able to detect E78 (NR1E) in the genome of *T. japonicus*. HR83 in the E5 group of subfamily NR2 was also not detected in *T. japonicus*, as reported for *D. pulex*, suggesting a crustacean-specific gene loss of these two genes. To clarify whether this gene loss was copepod-specific or not, we attempted to find E78 and HR83 in the genomes of other copepods (*T. californicus* and *P. nana*) but were not able to find any matches.

## Discussion

NRs are a diverse class of transcription factors that regulate hormonal and non-hormonal signalling processes in metazoans, and different numbers of NRs have been identified in different species [13–17]. Nuclear receptors have a conserved DBD and a moderately conserved LBD, which makes identification of nuclear receptors in the genome relatively easy and allows robust phylogenetic reconstruction at the superfamily level. Using whole genome and transcriptome sequence databases, we identified a total of 36 nuclear receptors, including isoforms, in the genome of the copepod *T. japonicus*.

### Homologous nuclear receptors involved in developmental processes

NRs have been reported to be good phylogenetic markers, as they give robust results because of their structural conservation [12]. Representatives of all seven subfamilies of nuclear receptors present in metazoans were identified in *T. japonicus*. Of 31 NRs in *T. japonicus*, 19 members were homologous to those in other species, while 12 receptors were unique to *T. japonicus* (Figure 2). Homologous nuclear receptors have been identified in *D. melanogaster* and *D. pulex* [17, 33]. Developmental functions of nuclear receptors in arthropods have been well studied in *Drosophila* [33]. Comparative functional studies of homologous NRs in other arthropods and expression studies of *T. japonicus* specific nuclear receptors might help clarify the involvement of nuclear receptors in development and ecdysone signaling in *T. japonicus*.

### Typical evolutionary patterns of NRs in crustaceans, arthropods, and chordates

Arthropod nuclear receptor subfamilies show different evolutionary patterns from those of chordates [12, 36]. Nuclear receptor members in groups A, B, and C of subfamily NR1 are present in *H. sapiens*, but not in arthropods. The same is true for SRs in the subfamilies NR3A and NR3C. There are two major SR lineages, namely the ER lineage and the AR/PR/GR/MR lineage that descended from a single ancestral receptor by genome duplication [37]. Despite the fact that SRs play important physiological roles in vertebrates and a few invertebrates (mollusks and annelids), homologues of these receptors have not been detected in arthropods. Many non-steroid receptors such as RXR, FTZ-F1, COUP-FT, and HNF4 appear to have been highly conserved through evolution, even though their developmental roles may be different in different metazoans [36, 38]. However, it appears that Ecdysozoa species do not have homologous members to NR3A in subfamily 3. *ERR* from *Drosophila* was identified as the gene most closely related to the vertebrate *ER* gene in a non-chordate species [39]. ERRs share a common ancestor with ERs based on phylogenetic analyses, and like TR and RAR, are present in Urbilateria [40]. SRs such as ER have well defined roles in mammalian reproduction. However, the ER may originally play a role in the development and regulation of the nervous system, which controls species-specific behavior and endocrine homeostasis in birds [40]. No ER has been found in arthropods (Figure 3).

### Expansion of NR1L members in crustaceans

**Figure 4:**
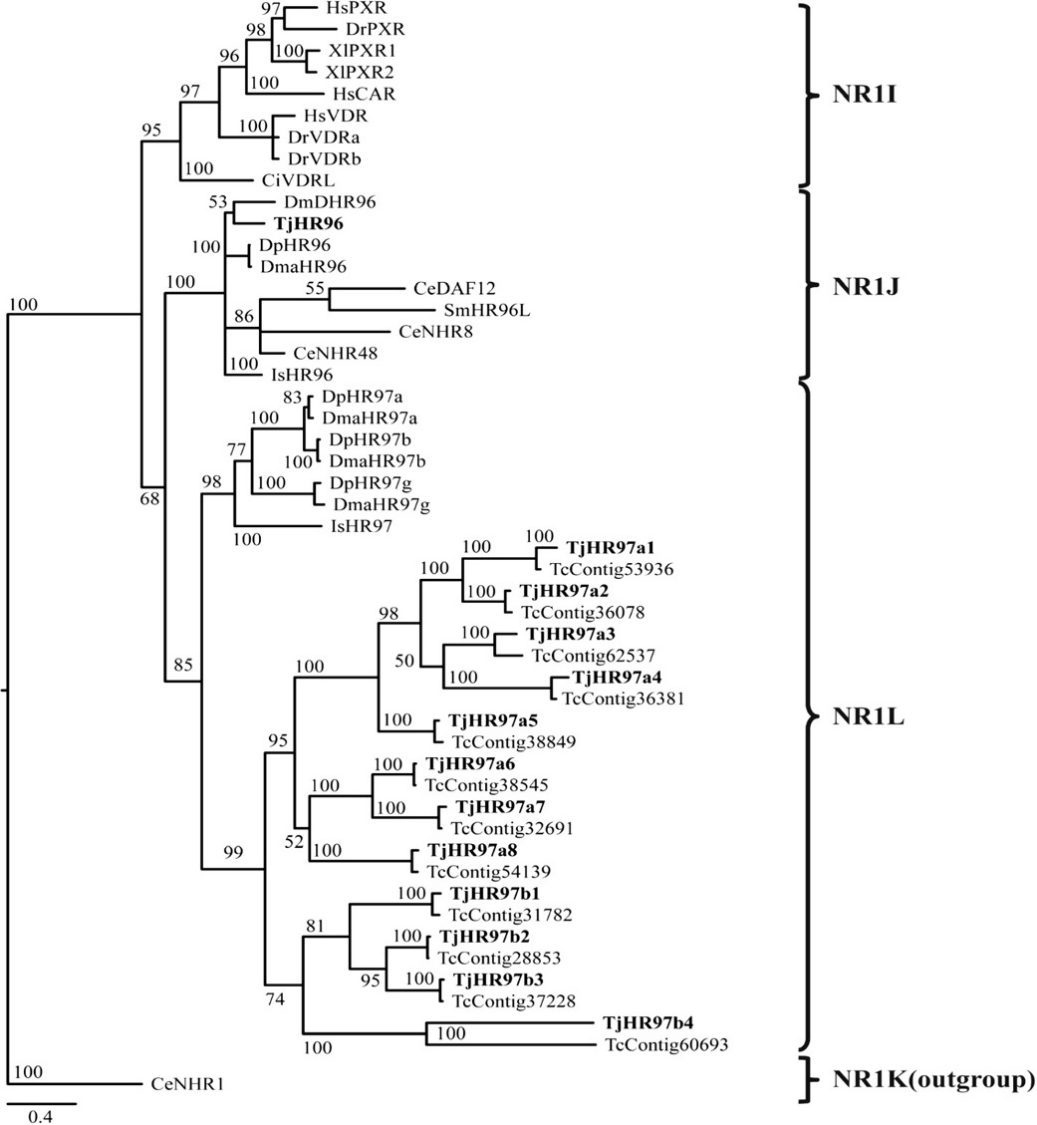
**Phylogenetic analysis of NR1I, NR1J, and NR1L in** ***T. japonicus*** **and other related species.** NR1K was used as an outgroup in this analysis. Details of model testing and parameters are provided in the Methods section. Tree is proportionally scaled, with the scale bar indicating sequence distance as number of substitutions. Species abbreviations: Ce: *Caenorhabditis elegans*, Ci: *Ciona intestinalis,* Dm: *Drosophila melanogaster*, Dma: *Daphnia magna*, Dp: *Daphnia pulex*, Dr: *Danio rerio*, Gg: *Gallus gallus*, Hs: *Homo sapiens*, Is: *Ixodes scapularis*, Sm: *Schistosoma mansoni*, Tc: *Tigriopus californicus,* Tj: *Tigriopus japonicus*, and Xl: *Xenopus laevis*.

Although the subfamily composition of all NR members in *T. japonicus* is similar to that of other arthropods, there are more NR1 subfamily members in the copepod than in *D. melanogaster* and *D. pulex*. In *T. japonicus*, 16 of 31 (52%) nuclear receptors are members of the NR1 subfamily. More than half (52%) of all nuclear receptors in another crustacean, *D. pulex*, are NR1 members [17]. Considering that 38% of NRs in *D. melanogaster* and 24% in *T. castaneum* are NR1 members [13, 16], crustaceans have more NR1 members than other arthropods. The unique NR1Ls that we identified support expansion of the NR1 subfamily in crustaceans. *Daphnia* has unique HR97/NR1Ls in subfamily NR1 (Figure 4) [17, 35]. We also detected 12 unique nuclear receptors in *T. japonicus*, which all clustered in subfamily NR1L based on phylogenetic analysis. Although we named these receptors ‘HR97/NR1L’ following the nomenclature for *Daphnia* species, the genetic distance between *T. japonicus* NR1L members and *Daphnia* NR1L subfamily members was high, implying that these receptors in *Daphnia* may be an independent lineage. More sequence information from copepod NRs needs to be obtained to study copepod-specific NR1L gene expansion.

Previously, lineage-specific expansions of 10 members of NR1H and three members of NR2E were demonstrated in the cephalochordate *Branchiostoma floridae* [41]. Moreover, a massive duplication of HNF4 (>250 HNF4-like NRs) occurred in *C. elegans* [42]. Also a lineage specific expansion in a Lophotrochozoan has also been identified recently in the NR1 subfamily (11 members) of the pacific oyster [43]. NR complements can therefore be shaped by lineage-specific loss and/or tandem duplication events. In most crustaceans, nuclear receptors involved in ecdysone signaling are well studied, but the entire NR superfamily has only been studied in *D. pulex* and *T. japonicus* thus far. The expansion of NR1L members identified in *T. japonicus* and copepods (*T. californicus* and *P. nana*) highlights the usefulness of studying copepods to gain a better understanding of comparative evolutionary diversification of NRs. Data from more diverse crustaceans would further increase our understanding of this expansion.

### Copepod-specific NR evolution

Although the overall pattern of nuclear receptor members present in *T. japonicus* is highly similar to that in other arthropods, we failed to detect a receptor homologous to E78 in *T. japonicus*, which was unexpected. In *Drosophila*, E75 and E78, orthologous of Rev-Erb, are derived from duplication events that did not occur in the vertebrate lineage [33]. Unlike other duplicated pairs of genes such as HR51 and HR83, HR39 and FTZ-F1, and DSF and TLL, the pair E75 and E78 was detected in *C. elegans*, indicating that this duplication event occurred before nematodes branched off from the arthropod lineage [33]. In addition, the E75-E78 pair was also identified in crustaceans [17]. Based on these observations, we expected that *T. japonicus* as a crustacean would have E75 and E78. However, we were not able to find E78, which suggests loss of this gene in this copepod lineage, because E78 is also absent from the genomes of *T. californicus* and *P. nana*. Further phylogenetic studies of E75 and E78 in more crustaceans and copepods species as well as other related organisms are required to resolve this issue.

Vertebrates do not have HR83 due to a gene loss event [42]. However, the HR83 gene has been identified in nematodes (NHR-239 in *C. elegans* and *C. briggsae*), an echinoderm (*Strongylocentrotus purpuratus*), and a hemichordate (*Saccoglossus kowalevskii*) [44]. In the red flour beetle *T. castaneum*, HR83 was the NR with the most divergent DBD and LBD domains, indicating decreased constraint on this gene in *T. castaneum* [4]. Particularly, the absence of an HR83 homologue in the genomes of *D. pulex*, *T. japonicus*, and other copepods (*T. californicus* and *P. nana*) suggests that the HR83 gene was present in the urbilaterian ancestor to the Arthropods but was subsequently selectively lost from the crustacean lineage. Loss of NHR-239/HR83 appears to be unique to crustaceans among invertebrates.

## Conclusions

To our knowledge, this is the first report of all members of the nuclear receptor superfamily in a copepod species. *T. japonicus* has 31 nuclear receptors in the genome, 12 of which are copepod-unique receptors. The overall pattern of nuclear receptor members in *T. japonicus* was highly similar to that in other arthropods, although there appears to have been copepod-specific expansion of NR1 subfamily members. The absence of E78 and HR83 from *T. japonicus* and two other copepods suggests the possibility that this is a copepod-specific gene loss.

## Methods

### Animal culture and maintenance

The copepod *T. japonicus* was maintained and reared in 0.2 μm-filtered seawater adjusted to 25°C with a photoperiod of 12 h:12 h light/dark and a salinity of 30 practical salinity units (psu). Copepods were fed with green algae (*Chlorella* sp.; approximately 6 × 10^4^ cells/mL). Species identification was based on morphological characteristics and sequence identity of the universal barcode marker, the mitochondrial cytochrome oxidase I (COI) gene [45].

### RNA extraction and cDNA synthesis

Approximately 300 adult copepods of both sexes were homogenized in three volumes of TRIZOL® reagent (Molecular Research Center, Inc., Cincinnati, OH, USA) with a tissue grinder and stored at -80°C until use. Total RNA was isolated from tissues according to the manufacturer’s instructions. Genomic DNA was removed using DNase I (Sigma, St. Louis, Mo, USA). Quantity of total RNA was measured at 230, 260, and 280 nm with a spectrophotometer (Ultrospec 2100 pro, Amersham Bioscience, Freiburg, Germany). To check for genomic DNA contamination, we loaded total RNA on a 1% agarose gel that contained ethidium bromide (EtBr) and visualized the gel after electrophoresis using a UV transilluminator (Wealtec Corp., Sparks, NV, USA). To verify total RNA quality, we loaded total RNA in a 1% formaldehyde/agarose gel stained with EtBr and checked 18/28S ribosomal RNAs integrity and band ratio. Single-strand cDNA was synthesized from total RNA using an oligo(dT)_20_ primer for reverse transcription (SuperScript™ III RT kit, Invitrogen, Carlsbad, CA, USA).

### Sequence retrieval and RACE of nuclear receptors

To obtain partial sequences of nuclear receptor cDNAs, we searched the *T. japonicus* genomic DNA database and RNA-Seq database. Briefly, the *T. japonicus* genome was sequenced with NGS technologies (GS-FLX titanium: 3,956,726 read no., 1,276,761,994 bp read length, 5.8 X coverage; Solexa shotgun: 217,004,832 read no., 16,492,367,232 bp read length, 75 X coverage; Solexa Mate Pair (5 kb): 210,606,298 read no., 7.792.433.026 bp read length, 35.4 X coverage). Accumulated read length was 25.56 giga base pairs (Gb) and total coverage was approximately 116 X. In assembly, NGS_Cell (ver. 4.01 beta 59916; CLC Bio., Boston, MA, USA) software was employed and a total of 60,979 scaffolds were constructed (174,022,895 bp scaffold length; average 2,854 bp; 6,355 bp N50). RNA-seq was accomplished using entire developmental stages including nauplius, copepodid, and adult. Trinity software was employed for RNA-seq *de novo* assembly [46]. A total of 59,983 mRNAs were annotated (78,311,238 bp mRNA length; 2,139 bp N50).

Contigs coding for nuclear receptor proteins obtained in this study were subjected to a BLAST analysis against the GenBank non-redundant (NR; including all GenBank, EMBL, DDBJ, and PDB sequence except EST, STS, GSS, or HTGS) amino acid sequence database to confirm sequence identities. All isolated genes were subjected to 5′- and 3′-Rapid Amplification of cDNA Ends (RACE) to obtain full-length transcripts according to the manufacturer’s protocol (Invitrogen). Primers were designed after comparing the exon/intron boundaries with genomic DNA using GENRUNNER software (Hastings Software, Inc., NY, USA), and confirmed with the Primer 3 program (Whitehead Institute for Biomedical Research, Cambridge, MA, USA). A series of RACE reactions were performed with target primers under the following conditions: 94°C/4 min; 40 cycles of 98°C/25s, 55°C/30s, 72°C/60s; and 72°C/10 min. Final PCR products were excised from 1% agarose/TBE gels, cloned into pCR2.1 TA vectors (Invitrogen), and sequenced with an ABI PRISM 3700 DNA analyzer (Bionics Co., Seoul, South Korea). All *T. japonicus* gene information has been registered in GenBank, and accession numbers of each gene are provided in Table 1.

### RT-PCR validation

To validate cDNA sequences of entire NRs identified in *T. japonicus*, RT-PCR was employed with two primers: a forward primer containing a start codon, and a reverse primer containing a stop codon. RT-PCR was conducted in a reaction mixture comprising 1 μl of first strand cDNA, 5 μl of 10× PCR reaction buffer, 1 μl of 10 mM dNTPs, 10 pM each primer, and 0.5 μl of NeoTherm™ *Taq* polymerase (GeneCraft, Köln, Germany). Reaction mixtures were subjected to amplification (1 cycle, 95°C, 5 min; 30 cycles, 94°C, 30 sec, 55°C, 30 sec, and 72°C, 30 sec; 1 cycle, 72°C, 7 min) using an iCycler (Bio-Rad, Hercules, CA, USA). The amplicon of each NR was loaded in an ethidium bromide (EtBr)-containing 1% agarose/TBE gel and visualized using a UV transilluminator (Wealtec Corp.) (Additional file 1: Figure S1).

### Annotation and phylogenetic analysis of nuclear receptors

Nomenclature of the nuclear receptors found in *T. japonicus* was determined based on sequence similarity and the results of phylogenetic analysis using the system recommended by the Nuclear Receptors Nomenclature Committee [47]. To investigate evolutionary relationships of *T. japonicus* NRs to other NRs, nuclear receptors identified in *T. japonicus* were subjected to phylogenetic analysis. We searched for NRs annotated in other species (*Caenorhabditis elegans*, *Daphnia pulex*, *Drosophila melanogaster*, *Danio rerio*, *Gallus gallus*, *Homo sapiens*, and *Xenopus laevis*) in NCBI (http://www.ncbi.nlm.nih.gov/projects/genome/seq/BlastGen/BlastGen.cgi?taxid=7070) by performing BLAST searches with each potential *T. japonicus* NR hit. Combined DBD-LBD amino acid sequences from *T. japonicus* and other species were aligned using MEGA software (ver. 6.0) with default parameters [48]. To establish the best-fit substitution model for phylogenetic analysis, the model with the lowest Bayesian Information Criterion (BIC) [49] and Akaike Information Criterion (AICc) [50, 51] scores was estimated using maximum likelihood (ML) analysis. According to the results of model test, WAG + G + I + F model was identified as the best-fit model and used in subsequent phylogenetic analyses. MrBayes (ver. 3.1.2) was used to reconstruct phylogenetic trees based on Bayesian inference [52]. The Markov chain Monte Carlo (MCMC) process was conducted with four chains and run for 5,000,000 generations. Sampling frequency was every 100 generations. After analysis, the first 5,000 trees were deleted as part of the burn-in process and a consensus tree of the remaining trees was constructed and visualized using SeaView ver. 4.2.1. Nodal support is reported as Bayesian posterior probabilities (maximum = 1.00). In addition, we estimated the genetic distance of the *T. japonicus* NR1L subfamily from the other NR1 subfamilies using a Dayhoff matrix-based model [53]. Rate variation among sites was modeled with a gamma distribution (shape parameter = 4). The analysis involved 49 amino acid sequences. All positions containing gaps and missing data were eliminated. There were a total of 60 positions in the final dataset. Evolutionary analyses were conducted in MEGA software (ver. 6.0) [48]. Nomenclature of each *T. japonicus* NR was based on the name of the homologous *D. pulex* and/or *D. melanogaster* NR after comparison of the evolutionary distance of the *T. japonicus* NR to that of the other ingroup members. To more specify the evolutionary relationship of the NR1L members, additional phylogenetic analysis was performed using only NR1 members in different taxa based on the same method described above (Additional file 1: Table S5).

### Availability of supporting data

Additional file 1. Supplementary tables and figures.

### Ethics statement

The copepod *T. japonicus* was reared in accordance with the guidelines of the Animal Welfare Ethics Committee of Sungkyunkwan University.

## Electronic supplementary material

Additional file 1: **Supplementary tables and figures.** (DOCX 548 KB)
