# Supplementary material for: Genome-wide identification of nuclear receptor (NR) superfamily genes in the copepod Tigriopus japonicus
Source: BMC Genomics. 2014 Nov 18;15(1):993. doi: 10.1186/1471-2164-15-993 (PMC4247118; doi:10.1186/1471-2164-15-993)
Supplement: Supplementary file 1 — Additional file 1: Supplementary tables and figures. (DOCX 548 KB) [file 12864_2014_6668_MOESM1_ESM.docx]

**Additional file 1**

**Supplementary Table S1. Sequences of primers used in RT-PCR validation.** Isotypes are indicated by ‘iso’.

| **Subfamilies** | **NR name** | **Oligo name** | **Sequence(5′→3′)** | **Nucleotide position(bp)** |
| --- | --- | --- | --- | --- |
| NR1 | TJ-E75-iso1 | TJ-NR055-F | AGCCCGCCCATCTTTAT | 5-21 |
|  |  | TJ-NR055-R | CTTCGGTGGGAATATCGTG | 2671-2689 |
|  | TJ-E75-iso2 | TJ-NR056-F | ATGGACCGTGAATGCTCC | 1-18 |
|  | TJ-E75-iso3 | TJ-NR057-F | AAGTCCGGCTTTACACTCC | 20-38 |
|  | TJ-HR3-iso1 | TJ-NR016-F | TCATCACGTGTGAAGGCTG | 155-173 |
|  |  | TJ-NR016-R | GACGGACACATCACCTTTGA | 1898-1917 |
|  | TJ-HR3-iso2 | TJ-NR017-F | GTCAATTCCTTGTTCTGCTCC | 4-24 |
|  | TJ-ECR | TJ-NR002-F | GTCTGGTAGTGCTCCACAAGTG | 3-24 |
|  |  | TJ-NR002-R | TTGATGTCCCAGATCTCGG | 1619-1637 |
|  | TJ-HR96 | TJ-NR036-F | GTATGCAAGGAAGAGACTGTGG | 16-37 |
|  |  | TJ-NR036-R | CAAATAACGCCGCAGGA | 1706-1722 |
|  | TJ-HR97a1 | TJ-NR001-F | TGGCGGTCAGAGTTGTTCT | 180-198 |
|  |  | TJ-NR001-R | GTATATCAATGGCCTCCTTGG | 1214-1234 |
|  | TJ-HR97a2 | TJ-NR007-F | CATGGATGAGGGAGTGTTTGA | 150-170 |
|  |  | TJ-NR007-R | TTGGGCCTCCCTAGCATAG | 1662-1680 |
|  | TJ-HR97a3 | TJ-NR022-F | GGAGACATTATTCCAAAGCG | 3-22 |
|  |  | TJ-NR022-R | GCTTCTCCCGTGATCATCA | 1409-1427 |
|  | TJ-HR97a4 | TJ-NR068-F | GATCATCATCATCTTCTCTTCAAATCC | 170-196 |
|  |  | TJ-NR068-R | TCAATTGGCGGTGTAATGAGTAAG | 1307-1330 |
|  | TJ-HR97a5 | TJ-NR024-F | AAGTGAAGACCTGGAAGAGACC | 219-240 |
|  |  | TJ-NR024-R | TCTTGTGTAGCTTGGATCTTGG | 1220-1241 |
|  | TJ-HR97a6 | TJ-NR031-F | ATGATGCTGGCTGATGGAC | 1-19 |
|  |  | TJ-NR031-R | CAAATGGTGAGCCGCTTAG | 1664-1682 |
|  | TJ-HR97a7 | TJ-NR064-F | AAACGGTCCAATCTCAATGCG | 103-123 |
|  |  | TJ-NR064-R | GAATAAGCTCTTCAGTGGGTGGAG | 1235-1258 |
|  | TJ-HR97a8 | TJ-NR051-F | AACTCATCGAGACTGTGAGGAG | 9-30 |
|  |  | TJ-NR051-R | TGAGGATCTCAGCGAGACATC | 1466-1486 |
|  | TJ-HR97b1 | TJ-NR030-F | GCCATCGTGTGTTTCTCCT | 151-169 |
|  |  | TJ-NR030-R | CCATACAGGCGATGAAGGTA | 1240-1259 |
|  | TJ-HR97b2 | TJ-NR041-F | GTTCCTACGTATTGCCAAGAC | 3-23 |
|  |  | TJ-NR041-R | GGGTCTCTTGCGTTTGATTAG | 1831-1851 |
|  | TJ-HR97b3 | TJ-NR050-F | CATGACGGACCATCTGGAT | 12-30 |
|  |  | TJ-NR050-R | TGGGATCGTATCGAAGGTG | 1679-1697 |
|  | TJ-HR97b4 | TJ-NR054-F | GTCCTAAGCCAGAAACAACCA | 29-49 |
|  |  | TJ-NR054-R | AGCGTTATACGTGTTGAGATCC | 2376-2397 |
| NR2 | TJ-HNF4 | TJ-NR066-F | CAACTACACATGGTGGGACCTACAG | 95-119 |
|  |  | TJ-NR066-R | GGTTAGACCTCGAGCATACGGATC | 1087-1110 |
|  | TJ-USP | TJ-NR006-F | TTGTTGAATGCGCCCTCT | 157-174 |
|  |  | TJ-NR006-R | GGTTCGGCAATACTCTTCC | 1128-1146 |
|  | TJ-HR78 | TJ-NR044-F | GAGTGTTTATCTGGCTCCACC | 15-35 |
|  |  | TJ-NR044-R | TCTCTTCTTCCATGGGCTCT | 2055-2074 |
|  | TJ-TLL | TJ-NR020-F | TCTTCGCCCAAGATTCCTC | 13-31 |
|  |  | TJ-NR020-R | GGCATATCTTCTCCTGACTTG | 1416-1436 |
|  | TJ-PNR | TJ-NR018-F | CGTCGGCGACTCATTTATC | 178-196 |
|  |  | TJ-NR018-R | GGACCACTCTCAATGTGACTTC | 1055-1076 |
|  | TJ-DSF | TJ-NR040-F | GGAACGGGTGACAGATTACTC | 4-24 |
|  |  | TJ-NR040-R | ACGAGGATATCGGTTGCG | 1405-1422 |
|  | TJ-SVP-iso1 | TJ-NR011-F | CGTATGGAGGGATCCAAGT | 27-45 |
|  |  | TJ-NR011-R | ATTGTGGCTGATGTAGCGG | 1259-1277 |
|  | TJ-SVP-iso2 | TJ-NR012-F | GGAGAACTCTTCTGTTTGGAGAG | 24-46 |
|  |  | TJ-NR012-R | ACATTGTGGCTGATGTAGCG | 1260-1279 |
| NR3 | TJ-ERR-iso1 | TJ-NR026-F | ACCAACAACTTTCCTGCCTC | 151-170 |
|  |  | TJ-NR026-R | TTTGCTGCTCTGAGCTTCC | 1584-1602 |
|  | TJ-ERR-iso2 | TJ-NR027-F | TCTCCTCGCATCCAGTTTG | 27-45 |
| NR4 | TJ-HR38 | TJ-NR067-F | GCATGGCCCTCAACAACATC | 318-337 |
|  |  | TJ-NR067-R | CGAGTTAATCACCTTGGATTGGAGG | 1551-1575 |
| NR5 | TJ-FTZ-F1 | TJ-NR032-F | CCATCTTGTCGCCCTAGAT | 9-27 |
|  |  | TJ-NR032-R | GGCGTGTAACATTTCCATGA | 1949-1968 |
|  | TJ-HR39 | TJ-NR063-F | GTGAGTGTGATGATGAGCGAG | 14-34 |
|  |  | TJ-NR063-R | TCCATCAGAAGGTTGAACCC | 2314-2333 |
| NR6 | TJ-HR4 | TJ-NR009-F | GCGGAAAGTGTGAACAACCT | 164-183 |
|  |  | TJ-NR009-R | CAAGCAAGCCAAGTAACGG | 1818-1836 |
| NR0 | TJ-KNR1 | TJ-NR059-F | TGATCTCAGAGTCCGGGAGT | 2-21 |
|  |  | TJ-NR059-R | ATGTTGGGCAGGCTCTGA | 1084-1101 |
|  | TJ-KNR2 | TJ-NR060-F | TGTCGCTCAAGGAGGATG | 2-19 |
|  |  | TJ-NR060-R | GGCGACAAGGGCAATACT | 738-755 |
|  | TJ-KNR3 | TJ-NR061-F | ATGTTCGGCTCCTCCAAC | 1-18 |
|  |  | TJ-NR061-R | CGTTAAATCCAGAGGCGTT | 1320-1338 |

**Supplementary Table S2. Domain sequence similarity of *T. japonicus* nuclear receptors to that of homologous nuclear receptors in related organisms.**

| Subfamily | *T. japonicus* (TJ) | *D. pulex* (Dp) | *D. melanogaster* (Dm) | *H. sapiens* (Hs) | Tj vs. Dp | Tj vs. Dm | Tj vs. Hs |
| --- | --- | --- | --- | --- | --- | --- | --- |
|  |  |  |  |  | DBD/LBD(%) | DBD/LBD(%) | DBD/LBD(%) |
| NR1 | TJ-E75 | DpE75 | E75 | Rev-ErbAα | 95/70 | 95/69 | 87/60 |
|  | TJ-HR3 | DpHR3 | DHR3 | RORβ | 98/74 | 96/69 | 90/51 |
|  | TJ-ECR | DpEcRa, DpEcRb | EcR | FXR, LXR | 76/53 | 71/74, 74/72 | 70/51, 58/62 |
|  | TJ-HR96 | DpHR96 | DHR96 | CAR,VDR,PXR | 85/80 | 88/65 | 62/32, 66/32, 54/27 |
|  | TJ-HR97a1 | DpHR97a | - | - | 64/20 | - | - |
|  | TJ-HR97a2 | DpHR97a | - | - | 66/19 | - | - |
|  | TJ-HR97a3 | DpHR97a | - | - | 61/9 | - | - |
|  | TJ-HR97a4 | DpHR97a | - | - | 61/28 | - | - |
|  | TJ-HR97a5 | DpHR97a | - | - | 63/12 | - | - |
|  | TJ-HR97a6 | DpHR97a | - | - | 50/29 | - | - |
|  | TJ-HR97a7 | DpHR97a | - | - | 62/25 | - | - |
|  | TJ-HR97a8 | DpHR97a | - | - | 61/32 | - | - |
|  | TJ-HR97b1 | DpHR97b | - | - | 64/35 | - | - |
|  | TJ-HR97b2 | DpHR97b | - | - | 70/37 | - | - |
|  | TJ-HR97b3 | DpHR97b | - | - | 58/35 | - | - |
|  | TJ-HR97b4 | DpHR97b | - | - | 48/22 | - | - |
| NR2 | TJ-HNF4 | DpHNF4 | HNF4 | HNF4α | 86/74 | 86/75 | 84/73 |
|  | TJ-USP | DpRXR | USP | RXRα | 89/71 | 90/51 | 89/69 |
|  | TJ-HR78 | DpHR78 | DHR78 | TR2,TR4 | 88/74 | 82/52 | 77/64, 77/69 |
|  | TJ-TLL | DpTLL | TLL | NR2E2 | 96/60 | 93/43 | 89/62 |
|  | TJ-PNR | DpPNR | PNR | PNR | 93/49 | 75/50 | 81/50 |
|  | TJ-DSF | DpDSF | DSF | TLX | 83/75 | 95/68 | 85/55 |
|  | TJ-SVP | DpSVP | SVP | COUP-TFI | 98/91 | 98/87 | 97/91 |
| NR3 | TJ-ERR | DpERR | ERR | ERRα | 94/65 | 91/59 | 91/57 |
| NR4 | TJ-HR38 | DpHR38 | DHR38 | NURR1 | 94/73 | 92/76 | 96/69 |
| NR5 | TJ-FTZ-F1 | DpFTZ-F1 | FTZ-F1 | LRH1, SF1 | 96/86 | 97/71 | 92/65, 89/57 |
|  | TJ-HR39 | DpHR39 | DHR39 | LRH1, SF1 | 96/96 | 93/93 | 69/17, 68/18 |
| NR6 | TJ-HR4 | DpHR4 | DHR4 | GCNF | 94/79 | 94/65 | 76/47 |
| NR0 | TJ-KNR1 | DpKNR-R1, DPKNR2 | KNI, KNRL, EGON | - | 89/- | 85/-, 90/- , 90/- | - |
|  | TJ-KNR2 | DpKNR-R1, DPKNR2 | KNI, KNRL, EGON | - | 89/- | 85/-, 91/- , 90/- | - |
|  | TJ-KNR3 | DpKNR-R1, DPKNR2 | KNI, KNRL, EGON | - | 89/- | 85/-, 88/- ,88/- | - |

**Supplementary Table S3. Sequence similarity (%) of the DNA-binding domain (DBD) and ligand-binding domain (LBD) of NR1L members unique to *T. japonicus*.**

|  | TJ-HR97a1 | TJ-HR97a2 | TJ-HR97a3 | TJ-HR97a4 | TJ-HR97a5 | TJ-HR97a6 | TJ-HR97a7 | TJ-HR97a8 | TJ-HR97b1 | TJ-HR97b2 | TJ-HR97b3 | TJ-HR97b4 |
| --- | --- | --- | --- | --- | --- | --- | --- | --- | --- | --- | --- | --- |
| TJ-HR97a1 |  | 66/59 | 59/26 | 61/25 | 61/41 | 50/13 | 64/31 | 62/18 | 58/20 | 60/16 | 60/15 | 50/14 |
| TJ-HR97a2 |  |  | 65/25 | 72/18 | 69/30 | 53/15 | 62/31 | 67/18 | 66/16 | 72/13 | 62/12 | 48/10 |
| TJ-HR97a3 |  |  |  | 72/11 | 67/37 | 50/9 | 57/22 | 57/10 | 60/9 | 63/7 | 60/6 | 46/7 |
| TJ-HR97a4 |  |  |  |  | 66/16 | 43/25 | 53/15 | 57/30 | 61/33 | 62/35 | 57/34 | 44/20 |
| TJ-HR97a5 |  |  |  |  |  | 49/13 | 56/28 | 60/15 | 58/13 | 60/15 | 64/12 | 51/10 |
| TJ-HR97a6 |  |  |  |  |  |  | 64/17 | 56/35 | 52/29 | 54/33 | 48/33 | 45/19 |
| TJ-HR97a7 |  |  |  |  |  |  |  | 72/19 | 66/21 | 70/16 | 63/19 | 52/15 |
| TJ-HR97a8 |  |  |  |  |  |  |  |  | 64/37 | 68/42 | 60/38 | 56/23 |
| TJ-HR97b1 |  |  |  |  |  |  |  |  |  | 74/53 | 70/52 | 57/15 |
| TJ-HR97b2 |  |  |  |  |  |  |  |  |  |  | 78/73 | 58/18 |
| TJ-HR97b3 |  |  |  |  |  |  |  |  |  |  |  | 57/18 |
| TJ-HR97b6 |  |  |  |  |  |  |  |  |  |  |  |  |

**Supplementary Table S4. Evolutionary distances for sequence pairs in the *T. japonicus* NR1L subfamily with other NR1 subfamilies.** Genetic distance is represented as the number of amino acid substitutions per site averaged over all sequence pairs between groups. Details of the calculations and parameters are provided in the Materials and Methods section. Gene information for NR1I, NR1J, and NR1L was collected from *Caenorhabditis elegans* (Ce), *Ciona intestinalis* (Ci), *Danio rerio* (Dr), *Daphnia magna* (Dma), *Daphnia pulex* (Dp), *Drosophila melanogaster* (Dm), *Homo sapiens* (Hs), *Ixodes scapularis* (Is), *Schistosoma mansoni* (Sm), *Tigriopus californicus* (Tc), and *Xenopus laevis* (Xl).

**Supplementary Table S5. NRs used for phylogenetic analysis of subfamily 1.**

|  | | |  |
| --- | --- | --- | --- |
| NR group | Receptor | Species (common name) | Accession No. |
| 1I | VDR | *H. sapiens*( Human) | NP_001017536 |
|  | VDR | *Danio rerio* | AAI62226 |
|  | VDR | *Danio rerio* | NP_001153457 |
|  | PXR | *H. sapiens*( Human) | AAD05436 |
|  | PXR | *Danio rerio* | NP_001092087 |
|  | PXR | *Xenopus laevis* | CAA53006 |
|  | PXR | *Xenopus laevis* | NP_001083606 |
|  | CAR | *H. sapiens*( Human) | AAY56401 |
|  | VDRL | *Ciona intestinalis* | NP_001071847 |
| 1J | HR96 | *Daphnia pulex* (D. pulex) | 442778 |
|  | HR96 | *Daphnia magna* | AEX93434 |
|  | HR96 | *Tigriopus japonicus(T.japonicus)* | KJ664194 |
|  | HR96 | *Ixodes scapularis* | XP_002404556 |
|  | DHR96 | *D. melanogaster* (fruitfly) | AAC46928 |
|  | DAF-12 | *Caenorhabditis elegans(C. elegans)* | AAD34462 |
|  | NHR-8 | *Caenorhabditis elegans(C. elegans)* | AAP31437 |
|  | NHR-48 | *Caenorhabditis elegans(C. elegans)* | CAD36502 |
|  | SmHR96L | *Schistosoma mansoni* | XP_002575014 |
| 1K | NHR-1 | *Caenorhabditis elegans(C. elegans)* | NP_001024855.1 |
| 1L | HR97a | *Daphnia pulex* (D. pulex) | 442812 |
|  | HR97a | *Daphnia magna* | JQ678702 |
|  | HR97b | *Daphnia pulex* (D. pulex) | 442724 |
|  | HR97b | *Daphnia magna* | JQ678703 |
|  | HR97g | *Daphnia pulex* (D. pulex) | 442655 |
|  | HR97g | *Daphnia magna* | JF792806 |
|  | HR97 | *Ixodes scapularis* | XP_002402961 |
|  | HR97a1 | *Tigriopus japonicus(T.japonicus)* | KJ664195 |
|  | HR97a2 | *Tigriopus japonicus(T.japonicus)* | KJ664196 |
|  | HR97a3 | *Tigriopus japonicus(T.japonicus)* | KJ664198 |
|  | HR97a4 | *Tigriopus japonicus(T.japonicus)* | KM676402 |
|  | HR97a5 | *Tigriopus japonicus(T.japonicus)* | KJ664199 |
|  | HR97a6 | *Tigriopus japonicus(T.japonicus)* | KJ664201 |
|  | HR97a7 | *Tigriopus japonicus(T.japonicus)* | KJ664206 |
|  | HR97a8 | *Tigriopus japonicus(T.japonicus)* | KJ664205 |
|  | HR97b1 | *Tigriopus japonicus(T.japonicus)* | KJ664200 |
|  | HR97b2 | *Tigriopus japonicus(T.japonicus)* | KJ664202 |
|  | HR97b3 | *Tigriopus japonicus(T.japonicus)* | KJ664203 |
|  | HR97b4 | *Tigriopus japonicus(T.japonicus)* | KJ664205 |
|  | contig53936 | *Tigriopus californicus* | JW538317.1 |
|  | contig36078 | *Tigriopus californicus* | JW510774.1 |
|  | contig62537 | *Tigriopus californicus* | JW536803.1 |
|  | contig36381 | *Tigriopus californicus* | JW510401.1 |
|  | contig38849 | *Tigriopus californicus* | JW503142.1 |
|  | contig31782 | *Tigriopus californicus* | JW538311.1 |
|  | contig28853 | *Tigriopus californicus* | JW536795.1 |
|  | contig37228 | *Tigriopus californicus* | JW508751.1 |
|  | contig38545 | *Tigriopus californicus* | JW504301.1 |
|  | contig32691 | *Tigriopus californicus* | JW539445.1 |
|  | contig54139 | *Tigriopus californicus* | JW510405.1 |
|  | contig60693 | *Tigriopus californicus* | JW511695.1 |

**Supplementary Figure S1. RT-PCR products encoding full-length nuclear receptors identified in the genome of *T. japonicus*.** ‘M’ in lane 1 of each figure indicates the 100 bp DNA ladder. Isotypes are identified by ‘iso’.

**FTZ-F1**


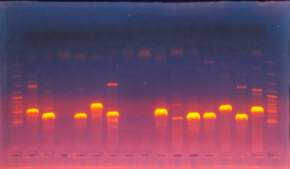

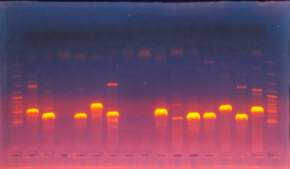

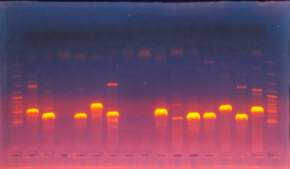

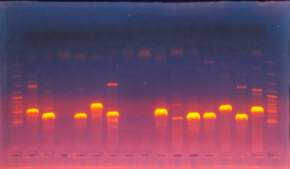


**HR97a1**


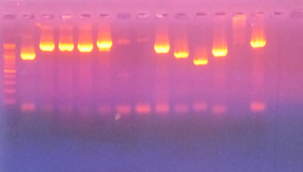

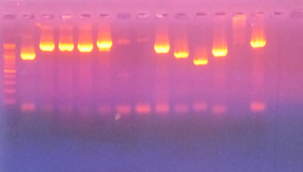

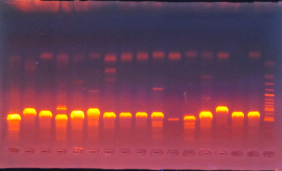

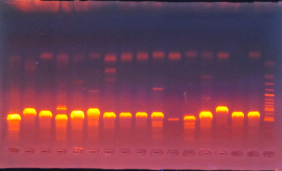


**ECR**

**USP**

**HR97a2**

**HR4**

**SVP-iso1**

**SVP-iso2**

**HR3-iso1**

**HR3-iso2**

**PNR**

**TLL**

**M**

**M**

**HR97a3**

**HR97a4**

**ERR iso1**

**ERR iso2**

**HR97b1**

**HR97b4**

**HR96**

**DSF**

**HR97b2**

**HR78**

**HR97b3**

**HR97b6**

**HR97g**

**E75a(iso1)**

**E75b(iso2)**

**M**

**E75c(iso3)**

**KNR1**

**KNR2**

**KNR3**

**HR39**

**HR38**

**HR97b5**

**HNF4**

**Supplementary Figure S2. Schematic diagram of the exon-intron structure of nuclear receptor isoforms in *T. japonicus*.** Translated regions of exons are shown as boxes. Exons are drawn to scale. Red, green, and yellow colored boxes denote the start codon of each NR isoform. Stop codon is marked with a vertical red line. DNA-binding domains (DBDs) and ligand-binding domains (LBDs) are marked in blue and gray, respectively. NR isotypes are indicated by ‘iso’.

**
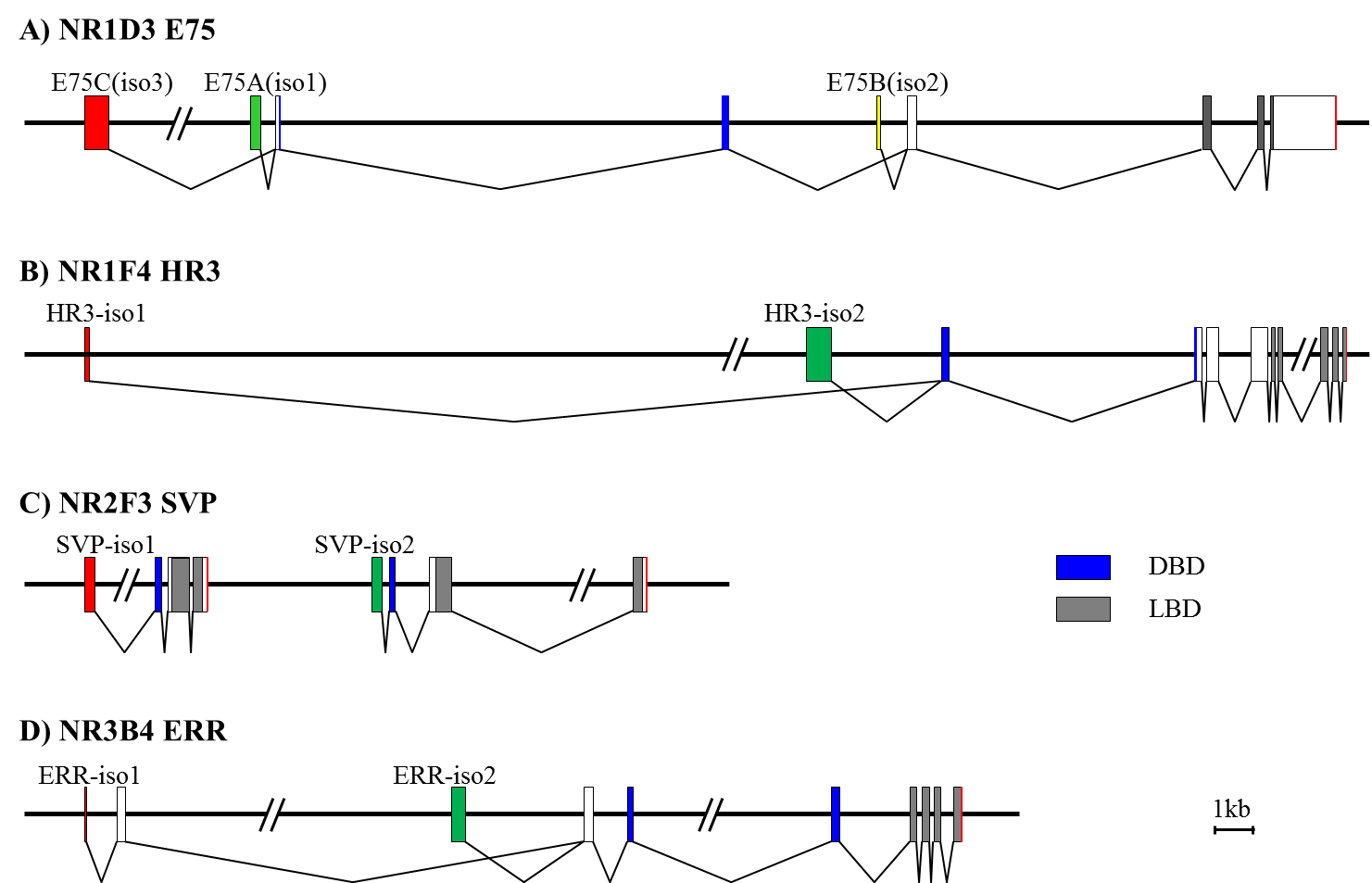
**
